# Case of Retention of Urine from Strictures, Relieved by Puncturing the Bladder, and Followed by Hæmaturia

**Published:** 1827-11

**Authors:** M. W. Andrews


### RETENTION OF URINE.

*Case of Retention of Urine from Strictures, relieved by puncturing the Bladder, and followed by Hæmaturia.* By M. W. Andrews, Esq.

J. P., a gentleman's servant, about forty-six years of age, had an attack of acute hepatitis. He was twice bled at the arm; leeches were applied over the region of the liver, which was followed by a blister, and mercurial purgatives with senna were administered. With this treatment the inflammatory symptoms were subdued, and in about ten days from the attack he was considered convalescent. During the time the blister was on, he suffered much from dysuria; which, however, was relieved by the use of mucilaginous drinks and diuretic medicines.

About ten days after, on Thursday, 14th June, he was attacked with pain and difficulty in voiding urine, passing only a very small quantity at a time, with an almost constant desire to make water:

the appearance of what he passed he described as resembling port wine. About twelve at night, he voided two ounces, with great straining; after which, it only came away by drops, attended with excruciating pain in the glans penis, until at length a complete retention took place.

I saw him first at ten A.M. Friday 15th June, and found him in a state of great local irritation, having passed a very restless night, with constant desire to make water, but was informed that none had passed since midnight. He was not feverish, nor did his pulse appear to be affected. He was very costive, not having had a motion for three days; and felt great pain in the urethra, particularly at the glans penis. The bladder, on examination, did not appear to contain much urine; nor did the pressure of the hand over the region of it give any increased uneasiness, or evince the least degree of tenderness.

I attempted to introduce a flexible gum catheter, which, from the spasm on the stricture, could not be passed beyond six inches. Under these circumstances, I did not think it right to use any violence, or force the catheter through the stricture, but considered it more prudent to evacuate the bowels, which I thought would probably be the means of relieving the bladder also. To effect which, I ordered him six grains of calomel, with half a grain of opium, in a pill, to be taken immediately; and in four hours after, a common senna draught, which operated copiously, but without producing any effect on the urinary organs. I then prescribed the following mixture:

℞. Spir. Æth. Nitros. fʒiij.; Mucil. Acaciæ f℥ij.; Tinct. Hyoscyam. fʒjss.; Spir. Juniperi comp. fʒij.; Aq. distillatæ f℥vss. M. fiat capiat cochl. iij. quartis horis.

In the evening, the pain at the glans penis and irritation in the urethra being increased, I made another attempt to pass the catheter, but could not advance it beyond six inches: hemorrhage followed. I then tried a small silver catheter, but with the same result. Having been foiled in my attempts to relieve the bladder, and being unwilling to increase the irritation by further trials at that time, and not considering an operation then justified, I passed down a fine plaster bougie as far as the stricture, secured it in the urethra, and ordered him again into the warm bath; which, with a full dose of opium, produced a profuse perspiration, and relieved the pain and irritation of the urethra.

The bath was ordered to be repeated at eight in the morning.

Saturday, 16th.—I learnt that he had passed some urine by the sides of the bougie during the night, which led me to hope that I had succeeded in removing the spasm: I therefore withdrew the bougie slowly, desiring him at the same time to make an effort to void his urine. A small quantity passed in a very fine stream, but not enough to afford relief. I then made another unsuccessful attempt to introduce the catheter, which was followed by a considerable discharge of blood. The bladder still not appearing to be much distended, and the pulse not indicating any general disturbance in the system, I determined on waiting a few hours longer, and, if I did not then succeed, to puncture it. I therefore introduced another bougie, and secured it in the urethra as before, and ordered him a full dose of opium, which produced much ease and a considerable perspiration. At ten in the evening, I put him again into the warm bath, and, after withdrawing the bougie, made another unsuccessful trial, which, like the former, was followed by a discharge of blood. Finding that the spasm could not be overcome, I punctured the bladder through the rectum, after a retention of forty-seven hours, and drew off thirty-two ounces of urine, which had a very healthy appearance. Having secured the canula in its situation, and fitted a small wooden plug to the orifice, I left him, with directions to the person who was to remain with him through the night to remove it whenever he felt a desire to make water.

Although there had apparently been so little disturbance in the system previous to the operation, the relief which he experienced from it was very great. I gave him a moderate dose of opium, and on Sunday morning (17th,) found him very tranquil, having passed a tolerably good night.

At ten A.M. I applied the caustic bougie to the stricture at six inches, which produced very little uneasiness, and completely removed the spasm; as at two P.M. he voided some urine through the urethra in a small stream, and continued to do so at intervals during the day and following night, a few drops only passing through the artificial channel. On Monday (18th), no water passing through the canula, I withdrew it, considering it no longer necessary, more especially as it began to produce a slight degree of irritation.

Tuesday, 19th.—At nine A.M. I again applied the caustic, and desired him to make water immediately after, which he did without the smallest difficulty, though in a fine stream.

At twelve o'clock he had a severe rigor, followed by considerable fever, which ended in profuse perspiration. This I at first considered likely to have been induced by the application, although there was no great degree of pain experienced at the time; but I afterwards had reason to attribute it to some derangement of the kidneys, as a total suppression of their secretion took place, and continued for thirty-six hours, during which period there was a great disposition to a comatose state. After emptying his bowels thoroughly by a mercurial purge, &c. I gave him the above diuretic mixture, as ordered on the 15th.

On Wednesday night (20th,) about thirty-six hours after the rigor, the secretion returned, and he passed, about midnight, a very small quantity (about half an ounce) of bloody urine. This he continued to do at intervals through the night and following day (Thursday 21st): the quantity gradually increasing, but always mixed with blood, in such proportions as at first sight to give the appearance of very high coloured porter, and always depositing a thin loose coagulum at the bottom of the vessel. Its smell was very offensive. This morning he had another distinct rigor, which did not last so long as the first, but was followed by an equal degree of fever and perspiration.

The water which passed during the stages of this paroxysm was less in quantity, contained a larger proportion of blood, and occasioned a considerable degree of heat and pain in passing through the urethra.

At night I gave him an anodyne, with one drachm of Spirit. Æth. Nitros.; and on the following day (Friday 22d,) a mixture containing two grains of the Sulphate of Quinine in each dose. This was repeated every four hours during the day, and the diuretic opiate at bedtime.

Saturday, 23d.—The quantity of urine voided was about two pints and a half in the twenty-four hours, but always mixed with blood. The tongue this morning was very much coated with a deep orange-coloured fur. I continued the mixture during the day, and gave him five grains of calomel at bedtime, and a senna draught on the following morning (Sunday 24th.) This medicine acted very well, and his tongue became much cleaner in consequence. The urine which passed during the night did not appear to be quite so bloody.

Continued the Quinine mixture, with the addition of f℥j. of Spir. Æth. Nitros. to each dose; and repeated the night draught.

Monday, 25th.—Passed a good night. Urine still containing a considerable quantity of blood, but passed without pain. Indeed, he did not now complain of pain any where, but great general debility, and no appetite.

Ordered him some white fish for dinner, and continued his medicines.

Tuesday, 26th.—Passed a good night. Urine contained rather more blood.

Omit the Quinine mixture, and ordered the following—℞. Potass. Nit. ʒss.; Mist. Amygd. f℥vj. M. fiat capiat quart. part. ter in die.—Repr H. Anod. diuret. et Mist. Amygd. nocte.

Continues to pass water without pain, though in a very small stream, which is sometimes divided, and still bloody. Tongue clean; pulse regular, and much stronger.

Fish dinner, with diluting drinks and farinaceous diet.—H. Anod. diuret. nocte.

Wednesday, 27th.—Passed a good night, but felt rather languid this morning. Urine of the same appearance; passes between four and five ounces at each time, once in about five or six hours; no pain in the region of the kidneys, or about the bladder. Bowels regularly moved once or twice every day. Ordered him a glass of wine and water at dinner, in consequence of being rather low; fish for dinner. Felt chilly and uncomfortable after taking the wine, and in the evening was very low and languid; pulse weak.

Continued the anodyne at bedtime.

Thursday, 28th.—Passed a good night, and felt stronger; pulse fuller and regular (seventy-two); urine appears to contain a greater proportion of blood.

℞. Ol. Terebinth. m. xxx.; Mucil. Acaciæ f℥jss.; Mist. Amygd. f℥vjss. M. capiat cochl. iij. ampl. quartis horis.—Repet^r H. Anod. diuret. h. s.

Friday, 29th.—Night good; bowels moved; pulse seventy-two; appetite somewhat improved; urine the same, but no pain in the kidneys, bladder, or urethra.

Cont^r medicamenta.

Saturday, 30th.—Slept well; feels stronger; tongue clean; pulse natural in number, but rather weak; urine in appearance as yesterday, but imparts a strong terebinthinate odour.

Continue same medicines.

Sunday, July 1st.—Pulse eighty; no alteration in the urine, and in other respects as yesterday.

Omit the Terebinthinate mixture.—℞. Liq. Potassæ f℥ij.; Mucil. Acaciæ f℥ij.; Tinct. Kino, f℥ij.; Aq. distillatæ f℥iijss. fiat M. ¼ part. ter in die sumend.—H. Anod. diuret. nocte.

Monday, 2d.—Passed a good night, and feels himself improving in strength. Urine rather clearer, with less blood.

Wednesday, 4th.—Still continuing to improve; urine less charged with blood; night very good; bowels rather confined.

Omit^r H. Anod. et sum^r H. Sennæ primo mane (which operated well). Cont^r Mist. Alkal.

Friday, 6th.—Urine perfectly free from any admixture of blood, but rather high coloured.

Cont^r med.

Sunday, 8th.—Urine in appearance and quantity natural.

Omit^r Mist. Alkal. et sumat^r Mist. Cinchonæ ter in die.

Monday, July 30th.—Health very much improved. Resumed the treatment of his strictures, which yielded to three more applications of the caustic, and allowed a flexible gum catheter to be freely passed into the bladder.

October 1st.—Continues perfectly well as regards his urinary organs, and has regained flesh and strength.

Query:—Was the first rigor induced by the caustic? or was it the consequence of the suppressed secretion of the kidneys? Did the blood come from the kidneys, or from the inner membrane of the bladder?

*Arlington-street; October*, 1827.

